# Modeling the Slow CD4+ T Cell Decline in HIV-Infected Individuals

**DOI:** 10.1371/journal.pcbi.1004665

**Published:** 2015-12-28

**Authors:** Sunpeng Wang, Patricia Hottz, Mauro Schechter, Libin Rong

**Affiliations:** 1 Department of Biology, New York University, New York, New York, United States of America; 2 Departamento de Medicina Preventiva, Faculdade de Medicina, Universidade Federal do Rio de Janeiro, Rio de Janeiro, Brazil; 3 Projeto Praça Onze, Hospital Escola São Francisco de Assis, Universidade Federal do Rio de Janeiro, Rio de Janeiro, Brazil; 4 Department of Mathematics and Statistics, and Center for Biomedical Research, Oakland University, Rochester, Michigan, United States of America; Imperial College London, UNITED KINGDOM

## Abstract

The progressive loss of CD4+ T cell population is the hallmark of HIV-1 infection but the mechanism underlying the slow T cell decline remains unclear. Some recent studies suggested that pyroptosis, a form of programmed cell death triggered during abortive HIV infection, is associated with the release of inflammatory cytokines, which can attract more CD4+ T cells to be infected. In this paper, we developed mathematical models to study whether this mechanism can explain the time scale of CD4+ T cell decline during HIV infection. Simulations of the models showed that cytokine induced T cell movement can explain the very slow decline of CD4+ T cells within untreated patients. The long-term CD4+ T cell dynamics predicted by the models were shown to be consistent with available data from patients in Rio de Janeiro, Brazil. Highly active antiretroviral therapy has the potential to restore the CD4+ T cell population but CD4+ response depends on the effectiveness of the therapy, when the therapy is initiated, and whether there are drug sanctuary sites. The model also showed that chronic inflammation induced by pyroptosis may facilitate persistence of the HIV latent reservoir by promoting homeostatic proliferation of memory CD4+ cells. These results improve our understanding of the long-term T cell dynamics in HIV-1 infection, and support that new treatment strategies, such as the use of caspase-1 inhibitors that inhibit pyroptosis, may maintain the CD4+ T cell population and reduce the latent reservoir size.

## Author Summary

The CD4+ T cell population within HIV-infected individuals declines slowly as disease progresses. When CD4+ cells drop to below 200 cells/ul, the infection is usually considered to enter the late stage, i.e., acquired immune deficiency syndrome (AIDS). CD4+ T cell depletion can take many years but the biological events underlying such slow decline are not well understood. Some studies showed that the majority of infected T cells in lymph nodes die by pyroptosis, a form of programmed cell death, which can release inflammatory signals attracting more CD4+ T cells to be infected. We developed mathematical models to describe this process and explored whether they can generate the long-term CD4+ T cell decline. We showed that pyroptosis induced cell movement can explain the slow time scale of CD4+ T cell depletion and that pyroptosis may also contribute to the persistence of latently infected cells, which represent a major obstacle to HIV eradication. The modeling prediction agrees with patient data in Rio de Janeiro, Brazil. These results suggest that a combination of current treatment regimens and caspase-1 inhibitor that can inhibit pyroptosis might provide a new way to maintain the CD4+ T cell population and eradicate the HIV latent reservoir.

## Introduction

HIV-1 progression to the AIDS stage within untreated patients usually takes many years. As HIV-1 infection progresses, the CD4+ T cell population declines slowly and the infected individual becomes progressively more susceptible to certain opportunistic infections and neoplasms. These are particularly common when CD4+ T cells reach a level below 200 cells/ul, which defines AIDS [1–7]. How HIV-1 infection induces progressive CD4+ T cell depletion is unclear [8]. One explanation is that the turnover rate of CD4+ T cells is significantly increased in HIV or simian immunodeficiency virus (SIV) infected subjects [9,10]. Therefore, massive activation of CD4+ T cells, which leads to more viral infection and cell death, might outrun the regeneration of T cells and cause progressive depletion. Another explanation is the failure of CD4+ memory T cell homeostasis during progressive HIV infection. This is possibly due to the destruction of the microenvironment of organs and tissues supporting T cell regeneration [3,11–14]. It remains unclear whether the impaired conformation of T cell regenerative tissues leads to the regeneration failure or it is merely a pathogenic reformation caused by HIV to promote viral replication.

Mathematical models may shed light on how the complex interplay between the immune response and viral infection leads to overt immunodeficiency. Matrajt et al. used a model to analyze the simian-human immunodeficiency virus (SHIV) infection data in macaques [15]. They found that uninfected or bystander cell death accounts for the majority of CD4+ T cell death [15]. Mohri et al. studied the turnover of CD4+ T cells and found that T cell depletion is primarily induced by increased cellular destruction rather than decreased cellular production [16]. Kovacs et al. also showed that HIV does not impair CD4+ T cell production but increases T cell proliferation [17]. Using a model including the activation of resting CD4+ T cells, Ribeiro et al. found that HIV infection increases both the activation rate of resting CD4+ T cells and the rates of death and proliferation of activated CD4+ T cells [18]. Chan et al. showed that the rapid proliferation of CD4+ T cells provides more targets for infection and that preservation of CD4+ T cells in natural host monkeys is due to the limited CD4+ T cell proliferation [19]. Thus, CD4+ T cell depletion may be caused by the massive immune activation during chronic infection. However, a model by Yates et al. suggested that if immune activation drives T cell decline, then the predicted decline would be very fast, which is not consistent with the time scale of T cell depletion during chronic infection [20]. The above observations and analyses may explain T cell depletion but the long-term dynamics of CD4+ T cells have been neither simulated by models nor compared with patient data. In a recent study, Hernandez-Vargas and Middleton [21] developed a model including the infection of macrophages to explain the three stages of HIV infection. Fast infection of CD4+ T cells can explain the CD4+ T cell and viral load dynamics in the early stages, while slow infection of macrophages may explain the dynamics in the advanced stages of infection. Whether macrophages form a long-term reservoir causing T cell depletion and viral explosion in the later stages of infection needs further experimental investigation.

Different from apoptosis, a programmed process that results in non-inflammatory cell death, pyroptosis is a form of programmed cell death associated with antimicrobial responses during inflammation [22]. During HIV infection, Doitsh et al. [23,24] found that when virus enters a CD4+ T cell that is non-permissive to viral infection, the caspase-1 pathway is triggered to induce pyroptosis, which can secrete inflammatory cytokines such as IL-1β. These cytokines establish a chronic inflammation state and attract more CD4+ T cells to the inflamed sites, resulting in more infection and cell death. Thus, pyroptosis generates a vicious cycle in which dying CD4+ T cells secrete inflammatory signals that attract more CD4+ T cells to be infected and die [23]. These findings suggest that HIV-1 may use the intrinsic feature of the immune system to seek targets of infection, establish productive viral replication, and meanwhile destroy the CD4+ T cell population.

Here we developed mathematical models incorporating the effect of pyroptosis to study whether it can explain the very slow T cell depletion during HIV-1 infection. Using the models we explored if highly active antiretroviral therapy (HAART) can preserve the CD4+ T cell population. We studied the effect of CD4+ T cell proliferation and CD8+ T cell response on CD4+ decline. We also compared our modeling prediction with clinical data obtained from patients in Rio de Janeiro, Brazil [25–28]. At last, we probed the possible contribution of chronic inflammation associated with pyroptosis to the HIV latent reservoir persistence.

## Methods

### Patient data

The patient data were obtained from seroconverters in 3 cohorts [25–28]. One cohort consists of high-risk, HIV-seronegative homosexual and bisexual men who did not report injection drug use, were enrolled between July 1995 and June 1998 and seroconverted during follow-up [26]. The other cohorts consist of seroconverters from high-risk HIV-seronegative homosexual and bisexual men patients who were enrolled from December 1998 to May 2001 in a study designed to evaluate the behavior impact of post-exposure prophylaxis [27], and participants from the control arm of SPARTAC, a randomized trial designed to evaluate the impact of short term antiretroviral therapy on the course of primary HIV infection [28]. The median of the CD4+ T cell data was derived from these cohort studies. The median of the Current Study Multicenter AIDS Cohort Study (MACS) was obtained from the study [29]. These patient data and medians were compared with modeling prediction.

### One-compartment model

Inflammatory cytokines released by abortively HIV-infected cells can attract more CD4+ T cells to be infected. In the following one-compartment model, to minimize the number of variables and parameters we described the effect of pyroptosis by use of an enhanced viral infection rate because of increased availability of CD4+ T cells attracted by cytokines to the inflamed sites.

$$\frac{dT}{dt}=\lambda -k\left(1+\gamma_{i}C\right)VT-d_{1}T$$

$$\frac{dT^{*}}{dt}=\left(1-f\right)k\left(1+\gamma_{i}C\right)VT-d_{2}T^{*}$$

$$\frac{dM^{*}}{dt}=fk\left(1+\gamma_{i}C\right)VT-d_{3}M^{*}$$

$$\frac{dV}{dt}=p_{v}T^{*}-d_{4}V$$

$$\frac{dC}{dt}=N_{c}d_{3}M^{*}-d_{5}C$$

**Fig 1:**
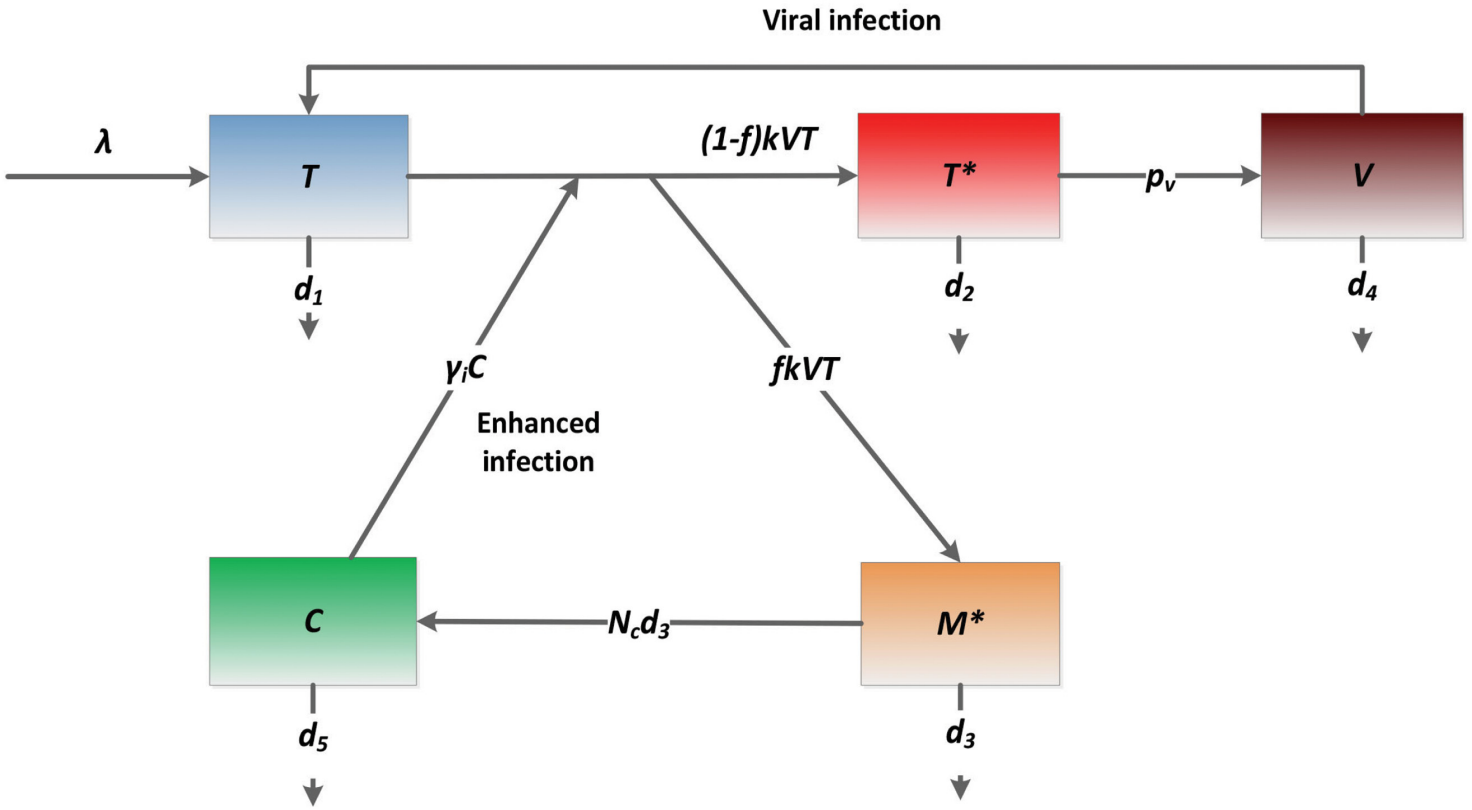
Schematic diagram of the one-compartment model. Inflammatory cytokines released during cell death by pyroptosis attract more CD4+ T cells (*T*) to be infected. The term *γ*_*i*_ *C·kVT* represents cytokine enhanced viral infection due to increased CD4+ T cell availability.

The variable *T* represents the population of uninfected CD4+ T cells. They are generated at the rate *λ*. Proliferation of target cells will be considered later. The infection rate is modeled by a mass action term *kVT*, which is enhanced by the inflammatory cytokine (*C*) with a factor *γ*_*i*_. Uninfected T cells die at a per capita rate *d*_*1*_. T* is the population of productively infected T cells and their death rate is *d*_*2*_. A fraction (*f*) of new infection is assumed to be abortively infected. The death rate of abortively infected T cells (M*) is *d*_*3*_. Virus (*V*) is generated by productively infected T cells with a viral production rate *p*_*v*_ and is cleared at a rate *d*_*4*_. Inflammatory cytokines are released with a burst size (*N*_*c*_) when an abortively infected cell dies. Thus, *N*_*c*_ *d*_*3*_ represents the generation rate of cytokines per abortively infected cell. The decay rate of cytokines is assumed to be *d*_*5*_. The schematic diagram of this model is shown in Fig 1. Parameters and values are listed in Table 1.

**Table 1:** A summary of all parameters and values used in the models.

Parameter	Description	Value	Ref.
*λ*	CD4+ T cell generation rate	/	Fitted
*k*	Viral infection rate	/	Fitted
*d*_*1*_	Death rate of uninfected CD4+ T cells	0.01 *day* ^*-1*^	[31]
*γ*_*i*_	Effectiveness of cytokines on infection	/	Fitted
*d*_*2*_	Death rate of infected CD4+ T cells	1 *day* ^*-1*^	[36]
*f*	Fraction of abortive infection	0.95	[23]
*d*_*3*_	Death rate of abortively infected cells	0.001 *day* ^*-1*^	[31]
*p*_*v*_	Viral production rate	/	Fitted
*d*_*4*_	Viral clearance rate	23 *day* ^*-1*^	[31]
*N*_*c*_	Burst size of cytokines	15 *molecule*	/
*d*_*5*_	Decay rate of cytokines	6.6 *day* ^-1^	[38]
*λ*_*1*_	Generation rate of CD4+ in blood	/	Fitted
*λ*_*2*_	Generation rate of CD4+ in lymph node	5×10^5^ *cell ml* ^*-1*^ *day* ^*-1*^	See text
*σ*_*1*_	CD4+ transfer rate to lymph node	0.01 *day* ^*-1*^	See text
*σ*_*2*_	CD4+ transfer rate to blood	0.0002 *day* ^*-1*^	See text
*γ*_*r*_	Effect of cytokines on CD4+ transportation	/	Fitted
*D*_*1*_	Rate of viral transfer to blood	0.1 *day* ^-1^	/
*D*_*2*_	Rate of viral transfer to lymph node	0.2 *day* ^-1^	/
*p*_*v1*_	Viral production rate in blood	/	Fitted
*p*_*v2*_	Viral production rate in lymph node	2000 *day* ^-1^	[32]
*ρ*	Hill coefficient in exponential function	2×10^−4^ *ml molecule* ^*-1*^	/
*α*	Killing rate of CD8+ T cells	0.01 *ml cell* ^*-1*^ *day* ^*-1*^	/
*p*_*E*_	Max activation rate of CD8+ T cells	100 *cells ml* ^*-1*^ *day* ^*-1*^	/
*θ*	Half max saturation of CD8+ activation	*50 cells ml* ^*-1*^	/
*η*	Effect of CD4+ T cells on CD8 activation	500 *cells/μl*	/
*d*_*E*_	Death rate of CD8+ T cells	0.06 *day* ^-1^	[62]
*μ*	Fraction of latent infection	0.001	[32]
*p*_*L*_	Proliferation of latently infected T cells	0.001 *day* ^-1^	See text
*φ*	Effect of cytokines on latent proliferation	10^−2^ *ml molecule* ^*-1*^	/
*L*_*max*_	Carrying capacity of latently infected cells	*100 cells ml* ^-1^	[31]
*d*_*L*_	Death rate of latently infected T cells	0.001 *day* ^-1^	[30]

In the above one-compartment model, we described the consequence of pyroptosis but did not explicitly model the cytokine-induced attraction of CD4+ T cells from elsewhere to the place where abortive infection occurs. Below we developed another model with two compartments to include cytokine-induced T cell movement explicitly. The model is more complicated and contains more parameters.

### Two-compartment model

$$\frac{dT_{1}}{dt}=\lambda_{1}-kV_{1}T_{1}-d_{1}T_{1}-\sigma_{1}\left(1+\gamma_{r}C\right)T_{1}+\sigma_{2}T_{2}$$

$$\frac{dT_{2}}{dt}=\lambda_{2}+\sigma_{1}\left(1+\gamma_{r}C\right)T_{1}-kV_{2}T_{2}-\sigma_{2}T_{2}-d_{1}T_{2}$$

$$\frac{dT_{1}^{*}}{dt}=kV_{1}T_{1}-d_{2}T_{1}^{*}$$

$$\frac{dT_{2}^{*}}{dt}=\left(1-f\right)kV_{2}T_{2}-d_{2}T_{2}^{*}$$

$$\frac{dM^{*}}{dt}=fkV_{2}T_{2}-d_{3}M^{*}$$

$$\frac{dV_{1}}{dt}=p_{v1}T_{1}^{*}-d_{4}V_{1}+D_{1}\left(V_{2}-V_{1}\right)$$

$$\frac{dV_{2}}{dt}=p_{v2}T_{2}^{*}-d_{4}V_{2}+D_{2}\left(V_{1}-V_{2}\right)$$

$$\frac{dC}{dt}=N_{C}d_{3}M^{*}-d_{5}C$$

**Fig 2:**
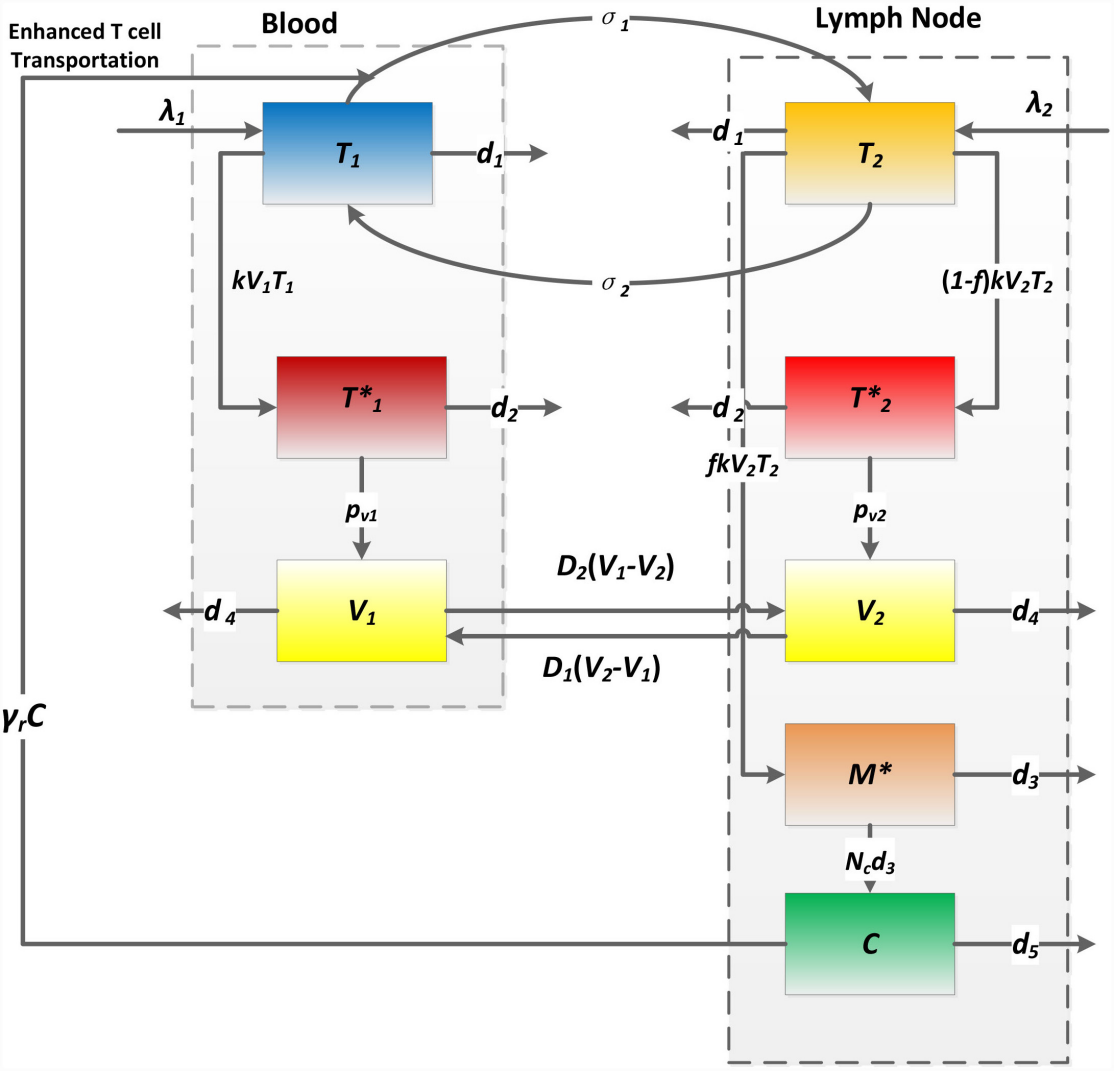
Schematic diagram of the two-compartment model. Parameters *σ*_*1*_ and *σ*_*2*_ represent the transportation rates of uninfected T cells between blood and lymph nodes. Cytokines (*C*) attract uninfected T cells (*T*_*1*_) from blood into lymph nodes at an enhanced rate *γ*_*r*_ *C*. Parameters *D*_*1*_ and *D*_*2*_ represent the transportation rates of virus between two compartments.

In the model there are two compartments: one represents the blood (*T*_*1*_) and the other represents human lymphoid tissues (*T*_*2*_) such as lymph nodes in which abortive infection takes place on a large scale [23]. CD4+ T cells in compartment I (or II) can transport to compartment II (or I) at a rate *σ*_1_ (or *σ*_2_). In blood, cytokines released during abortive infection cannot accumulate as in lymphoid tissues. They cannot attract other immune cells to fight the infection and contribute to inflammation. Thus, pyroptosis is assumed to take place only in lymphoid tissues (compartment II), as observed in ref. [23]. The transportation rate *σ*_1_ from the blood to tissues is assumed to be enhanced by a factor (1+*γ*_*r*_ *C*) due to inflammatory cytokines (*C*) released during pyroptosis in compartment II. Viruses (*V*_*1*_ and *V*_*2*_) can also transport between two compartments with the rates *D*_*2*_(*V*_1_-*V*_2_) and *D*_*1*_(*V*_2_-*V*_1_), which depend on the difference of viral load in the two compartments. Because the dynamics of the virus are much faster than those of infected cells, it is reasonable to assume that they are proportional to each other. Thus, we only included the transportation of virus between compartments. In the Supporting Information (S1 Text and S7 Fig), we added the transportation of infected cells to the model and found that the model prediction is similar to the case without infected cell transportation. All the other variables and parameters (summarized in Table 1) can be defined similarly as those in the one-compartment model (Fig 1). The schematic diagram of the two-compartment model is shown in Fig 2.

### Model parameters

For model simulation, we fixed most of parameters based on existing experimental data and our previous modeling studies [30–33]. Because the CD4+ T cell level within an uninfected individual ranges normally from 500 cells/μl to 1500 cells/μl, we changed the unit to cells/ml and assumed CD4+ T cells to be 10^6^ cells/ml before infection [34]. The death rate (*d*_1_) of uninfected CD4+ T cells is assumed to be 0.01 day^-1^ [35]. Thus, from the steady state of target cells before infection, we obtained that the generation rate (*λ*) of target cells is 10^6^(0.01) = 10^4^ cells ml^-1^ day^-1^. The viral infection rate *k* is assumed to be 2.4×10^−8^ ml virion^-1^ day^-1^ [30]. The death rate of infected T cells is *d*_2_ = 1 day^-1^ [36]. We chose the parameter *γ*_*i*_ to be 2×10^−4^ ml molecule^-1^. The viral production rate of productively infected T cells in the one-compartment model is chosen to be 2.5×10^4^ virions cell^-1^ day^-1^ [37]. As described by Doitsh et al. [23,24], abortive infection accounts for 95% of the total infection. Thus, we chose *f* to be 0.95. Because abortive infection mainly takes place in non-permissive quiescent T cells, we chose their death rate (*d*_*3*_) to be 0.001 day^-1^ [31,32]. The burst size of cytokines is fixed to *N*_*c*_ = 15 molecules. The half-life of IL-1β is about 2.5 hours [38]. Thus, we chose the decay rate of cytokines to be *d*_5_ = 6.6 day^-1^. We also performed sensitivity tests of the modeling prediction on a number of parameters.

### Data fitting

We fit both the one-compartment and two-compartment models to subjects with more than 10 data points [25–29]. The root mean square (RMS) between model prediction and patient data is minimized for each patient. RMS is calculated using the following formula

$$RMS=\sqrt{\frac{\Sigma_{i=1}^{n}{\left(T\left(t_{i}\right)+T^{*}\left(t_{i}\right)-\hat{T}\left(t_{i}\right)\right)}^{2}}{n}}$$

where *T*(*t*_*i*_)+T*(*t*_*i*_) represents the CD4+ T cell population level in blood at time *t*_*i*_ predicted by the model,$\;\hat{T}(t_{i})$ is the corresponding patient data at *t*_*i*_. We used *T*_*1*_(*t*_*i*_)+*T*_*1*_ *(*t*_*i*_) in the fitting for the two-compartment model. Parameter estimates are based on the best fit that achieves the minimum RMS. Data fitting is performed using the R programming language.

### Model comparison by AIC

In order to statistically compare the best fits of using the two models, we calculated the Akaike information criterion (AIC). The model with a lower AIC value fits the data better from a statistical viewpoint. The AIC is calculated using the following formula

$$AIC=n\text{ln}\left(\frac{RSS}{n}\right)+2m$$

$$RSS=\sum_{i=1}^{n}{\left(T\left(t_{i}\right)+T^{*}\left(t_{i}\right)-\hat{T}\left(t_{i}\right)\right)}^{2}$$

where *n* is the number of observations (i.e. number of data points) and *m* is the number of fitted parameters. RSS is the residual sum of squares. *T*(*t*_*i*_), T*(*t*_*i*_) and $\hat{T}(t_{i})$ are the same as those defined in the calculation of RMS.

### Confidence interval

We obtained the 95% confidence intervals for fitted parameters using a bootstrap method [39], where the residuals to the best fit were re-sampled 200 times.

## Results

### Slow depletion of CD4+ T cells

**Fig 3:**
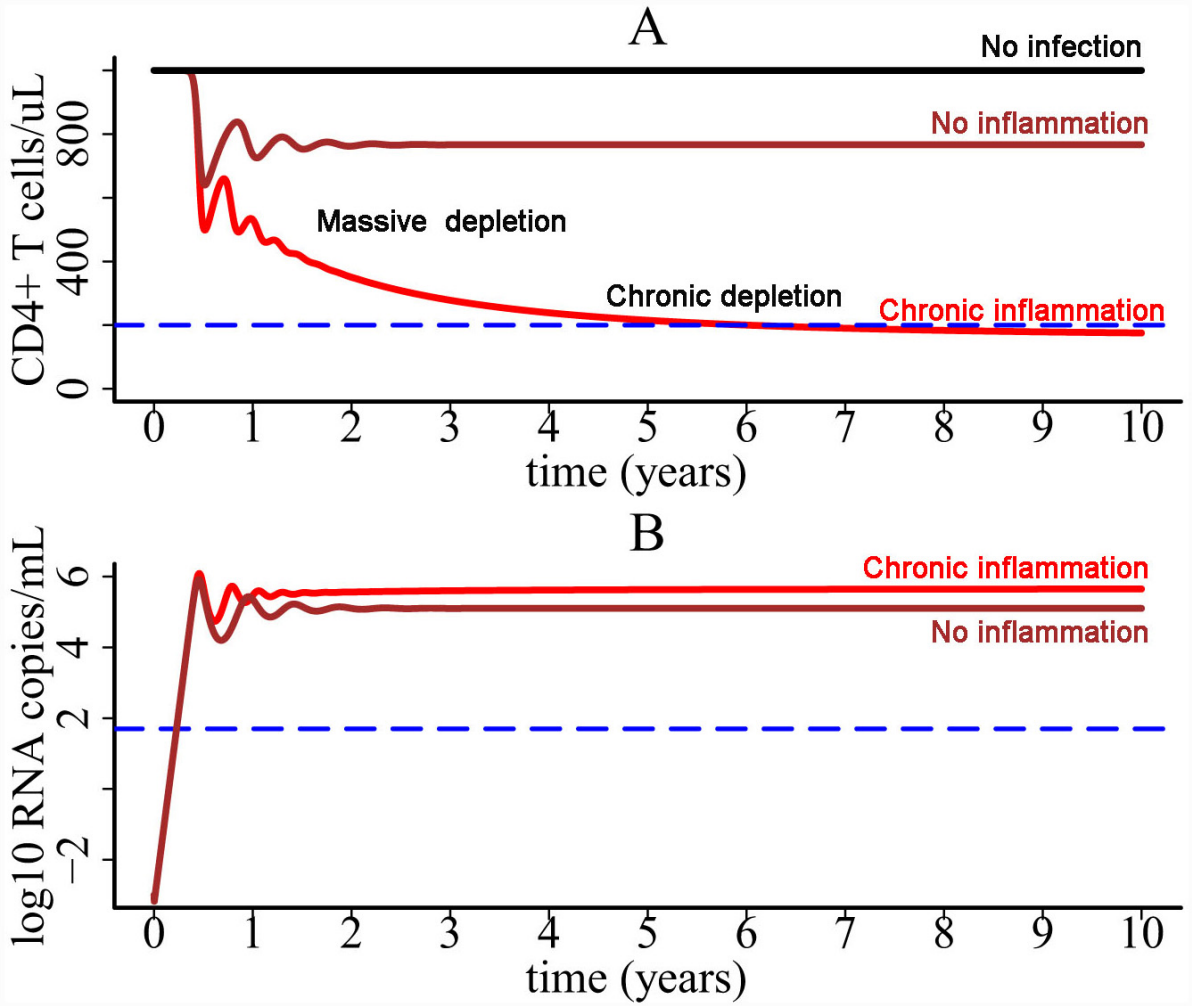
CD4+ T cell decline and viral load dynamics predicted by the one-compartment model. (A) CD4+ T cell decline consists of two major phases. A rapid and massive decline is caused by enormous viral infection during the early stage. It is followed by a progressive depletion phase, which is driven by pyroptosis enhanced viral infection. (B) Viral load dynamics generated by the one-compartment model with and without chronic inflammation.

Using the parameter values listed in Methods and initial values *V*(0) = 1×10^−3^ RNA copies/ml, *T*(0) = 10^3^ cells/μl, T*(0) = 0, M*(0) = 0, and *C*(0) = 0 in the one-compartment model, we showed that the population of CD4+ count declines from 10^3^ cells/μl to about 200 cells/μl around the 6^th^ year after infection (Fig 3A). This is consistent with the slow time scale of T cell decline during HIV infection. The entire T cell depletion course consists of two major phases. The first massive depletion phase is rapid, followed by a slower chronic depletion phase (Fig 3A). The first-phase T cell decline is due to the substantial viral infection during the early stage. If there is no infection (*k =* 0), then the T cell level would stabilize at the initial level (Fig 3A). The slow second-phase T cell decline is due to pyroptosis enhanced viral infection. Without the effect of inflammatory cytokines released during pyroptosis (i.e. *γ*_*i*_ = 0 or no inflammation in Fig 3A), a balance between T cell generation and viral infection is reached and the T cell population is maintained at a steady state level. This agrees with the prediction of most viral dynamics models without treatment. Because of pyroptosis, cytokine-enhanced viral infection breaks the balance between cellular production and viral infection, which makes the T cell level decline at a very low rate and approach the immune-deficient level after several years (Fig 3A).

The viral load change was plotted in Fig 3B. Without the effect of inflammatory cytokines, the viral load reaches a steady state level. When there is cytokine enhanced viral infection, viral load increases very slowly during the phase of chronic infection (Fig 3B).

Using a constant *λ* is a simple way to approximate the generation of target cells. We included the proliferation of target cells in the model (S1 Text). Simulation with different proliferation rates is shown in S1 Fig. As the proliferation rate increases, the decline of CD4+ T cells becomes faster. This is because more target cells lead to more abortive infection, which releases more cytokines attracting more CD4+ T cells to be infected and die. This prediction is consistent with the observation that the level of T cell proliferation in non-pathogenic infection (e.g. SIV infection in natural host monkeys such as sooty mangabeys or mandrills that do not develop AIDS-like diseases) was much lower than in pathogenic infection, e.g., SIV in rhesus macaques [40,41]. This provides an additional support to the view that an attenuated rather than effective adaptive immune response preserves immune function in natural host monkeys [42].

**Fig 4:**
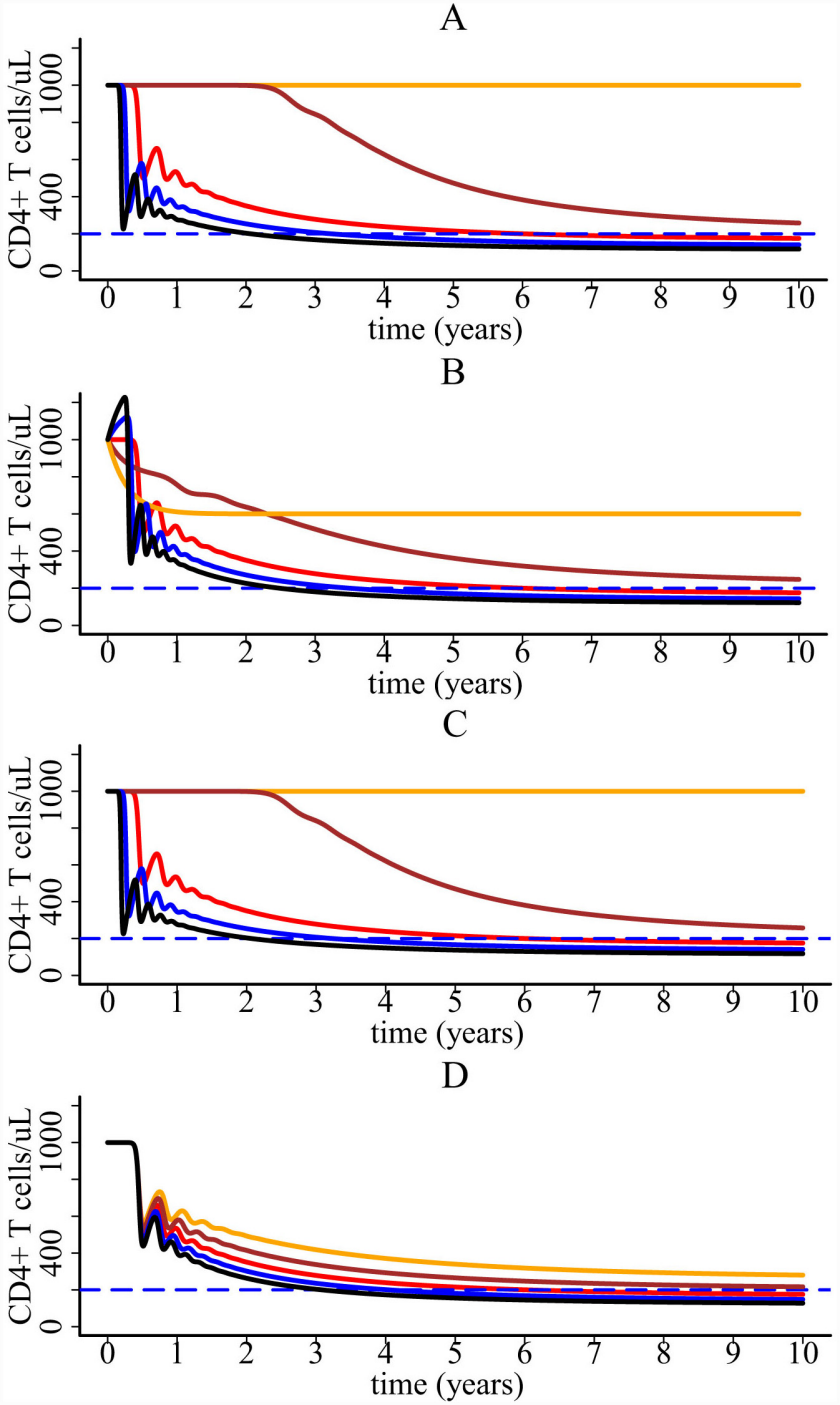
CD4+ T cell dynamics predicted by the one-compartment model with different parameter values. (A) Sensitivity test on the infection rate *k*. The value of *k* increases from 1.44×10^−8^ (orange line) to 3.36 ×10^−8^ ml virion^-1^ day^-1^ (black line) with four equal increments. (B) Sensitivity test on the target cell generate rate *λ*. The value of *λ* increases from 6000 (orange line) to 14000 cell ml^-1^day^-1^ (black line) with four equal increments. (C) Sensitivity test on the viral production rate *p*_*v*_. The value of *p*_*v*_ increases from 15000 (orange line) to 35000 virions cell^-1^ day^-1^ (black line) with four equal increments. (D) Sensitivity test on *γ*_*i*_, the effect of cytokines on infection. The value of *γ*_*i*_ increases from 1.2×10^−4^ (orange line) to 2.8 ×10^−4^ ml molecule^-1^ (black line) with four equal increments.

We performed sensitivity analysis of the CD4+ T cell decline for a number of parameters. Fig 4 shows that the sensitivity tests on parameters *k*, *λ*, *p*_*v*_ and *γ*_*i*_. S2–S5 Figs show the tests on parameters *N*_*c*_, *d*_*3*_, *d*_*5*_, and *f*, respectively. We found that the model is robust in generating the slow decline of CD4+ T cells, although the model prediction is more sensitive to three parameters *k*, *p*_*v*_ and *f* (see Figs 4A, 4C and S5).

In the above simulation, we assumed that the viral infection enhancement parameter *γ*_*i*_ is a constant. When the concentration of inflammatory cytokines is low, they may not be able to trigger the attraction of CD4+ T cells from elsewhere. Thus, we simulated a scenario in which enhanced viral infection is triggered only when the level of cytokines is above a threshold value. We chose *γ*_*i*_ to be the following step function. It is zero if the level of cytokines is below a certain level.

$$\gamma_{i}\left(C\right)=\{\begin{array}{ll} 0, & C<threshold\\ \gamma_{i}>0, & C\geq threshold \end{array}$$

**Fig 5:**
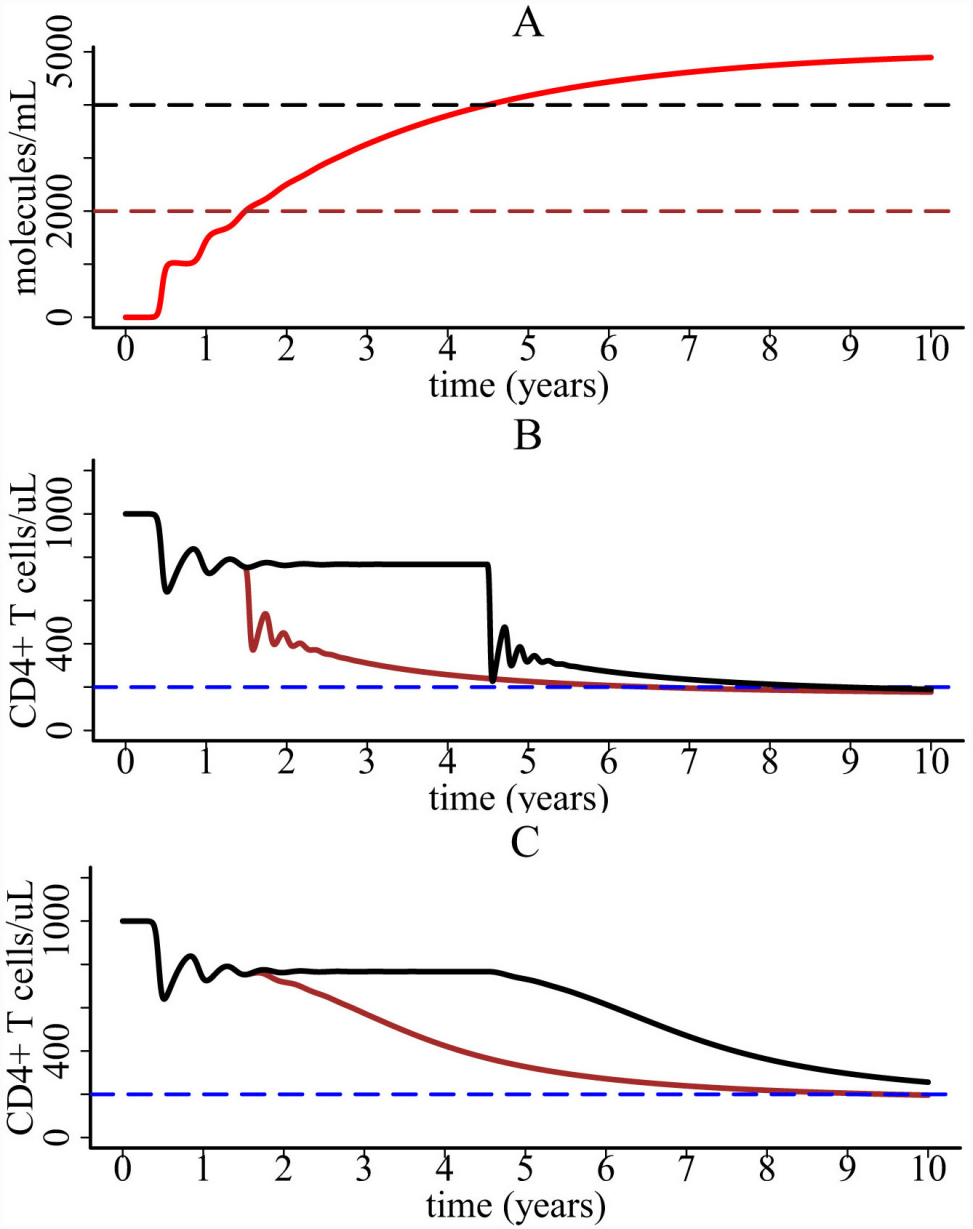
Simulation with a concentration-dependent cytokine enhanced function *γ*_*i*_. (A) The dynamics of cytokines. The black and brown dashed lines represent two threshold values we chose in simulation. (B) CD4+ T cell dynamics generated by the one-compartment model with a step function for the parameter *γ*_*i*_. The brown line corresponds to the threshold of 2000 molecules/ml and the black corresponds to the threshold of 4000 molecules/ml. (C) CD4+ T cell dynamics generated by the one-compartment model when the parameter *γ*_*i*_ is an exponential function of the concentration of cytokines.

The threshold value was chosen to be 2000 or 4000 molecules/ml in Fig 5A. CD4+ T cells do not decline until the level of cytokines reaches the corresponding threshold (Fig 5B).

A more realistic scenario is that *γ*_*i*_ increases gradually when the concentration of cytokines is above the threshold. We chose *γ*_*i*_(*C*) to be the following exponential function.

$$\gamma_{i}\left(C\right)=\{\begin{array}{ll} 0, & C<threshold\\ \gamma_{i}(1-e^{-\rho \left(C-threshold\right)}), & C\geq threshold \end{array}$$

The hill coefficient *ρ* determines how fast *γ*_*i*_(*C*) increases from 0 to its maximum value *γ*_*i*_. Both *ρ* and *γ*_*i*_ were fixed to 2×10^−4^ ml molecule^-1^. With a non-constant parameter *γ*_*i*_(*C*), we found that CD4+ T cells also undergo a slow decline to below 200 cells/μl (Fig 5B and 5C). Using an exponential function for *γ*_*i*_(*C*), the decline of CD4+ T cells is smoother than the case using a step function.

### Influence of HAART

**Fig 6:**
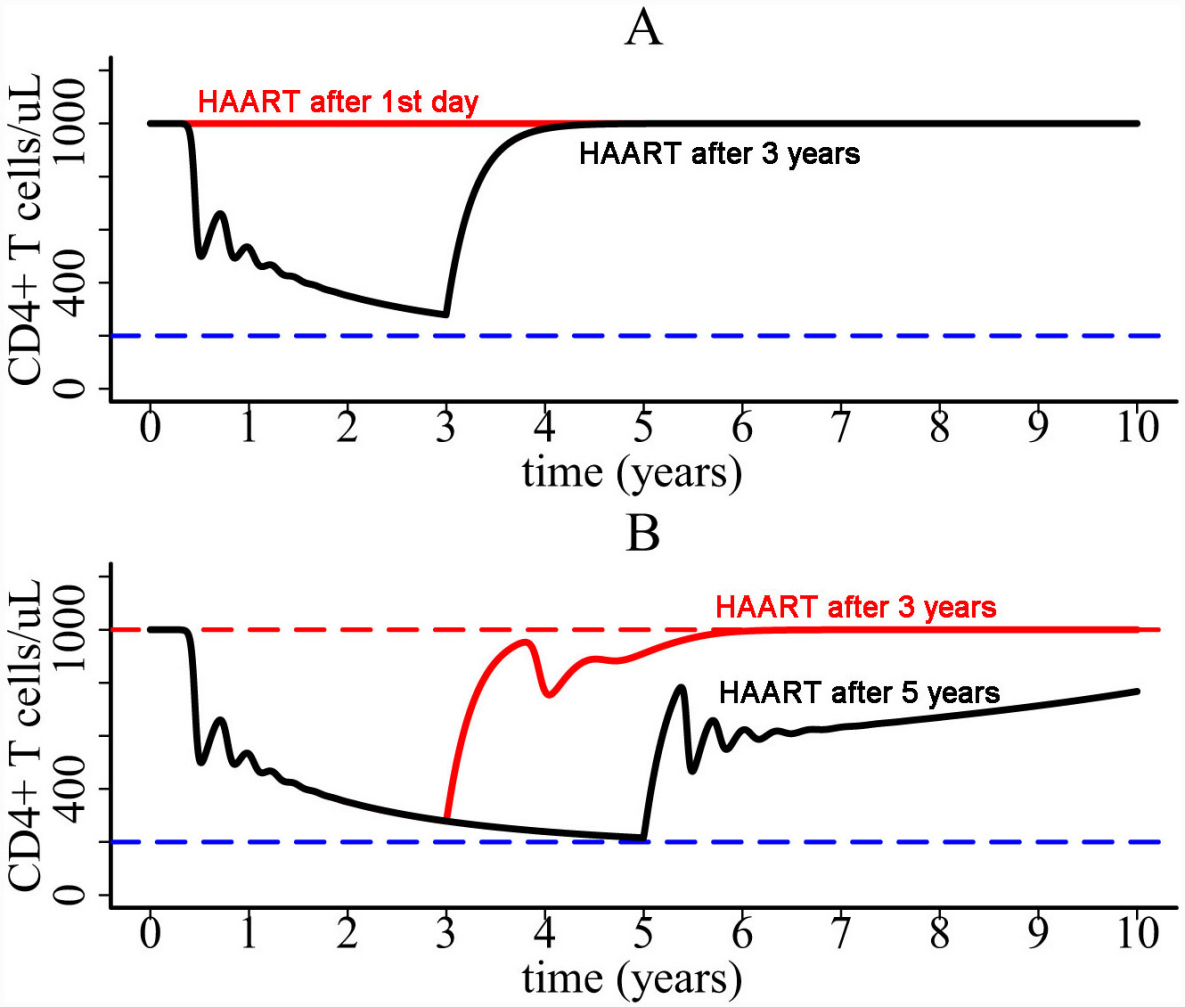
Influence of HAART on CD4+ T cell dynamics. (A) If treatment is very effective, then the CD4+ T cell population is predicted to be restored. (B) If drug efficacy is lower, then the later HAART is initiated the longer it takes for the T cell population to increase.

Using the one-compartment model, we studied if HAART can rescue the CD4+ T cell population. During HAART we assumed that the viral infection rate *k* is reduced by a factor (1-*ε*), where *ε* is the overall drug efficacy of the treatment [32]. The simulation shows that if the treatment effectiveness is very high, then CD4+ count can rebound to its pre-infection level (Fig 6A) no matter when HAART is initiated. For lower treatment effectiveness (e.g. *ε* = 0.6 in Fig 6B), the patient needs a relatively long time to restore the CD4+ T cell population. The later HAART starts, the longer it takes for CD4+ T cell restoration (Fig 6B). When the treatment effectiveness is further lower, CD4+ T cell depletion could not be prevented. These results suggest that HAART has the potential to rescue CD4+ T cell population, but CD4+ response depends on the effectiveness of the therapy and when the therapy is initiated.

### Model with CD8+ T cell response

We included CD8+ T cells in the one-compartment model to study the interaction between CD4+ T cell decline and CD8+ T cell response. CD8+ T cells (*E*) are assumed to kill infected T cells at a rate αET*. The activation rate of CD8+ T cells depends on the level of infected cells with a half-maximal saturation constant *θ*. *p*_*E*_ is the maximum activation rate. CD4+ T cells play an important role in activating the adaptive immune response. We used another saturation function *T*/(*T*+*η*) to account for this influence. The T* and *E* equations are given below.

$$\frac{dT^{*}}{dt}=\left(1-f\right)k\left(1+\gamma_{i}C\right)VT-d_{2}T^{*}-\alpha ET^{*}$$

$$\frac{dE}{dt}=p_{E}\left(\frac{T^{*}}{T^{*}+\theta }\right)\left(\frac{T}{T+\eta }\right)-d_{E}E$$

**Fig 7:**
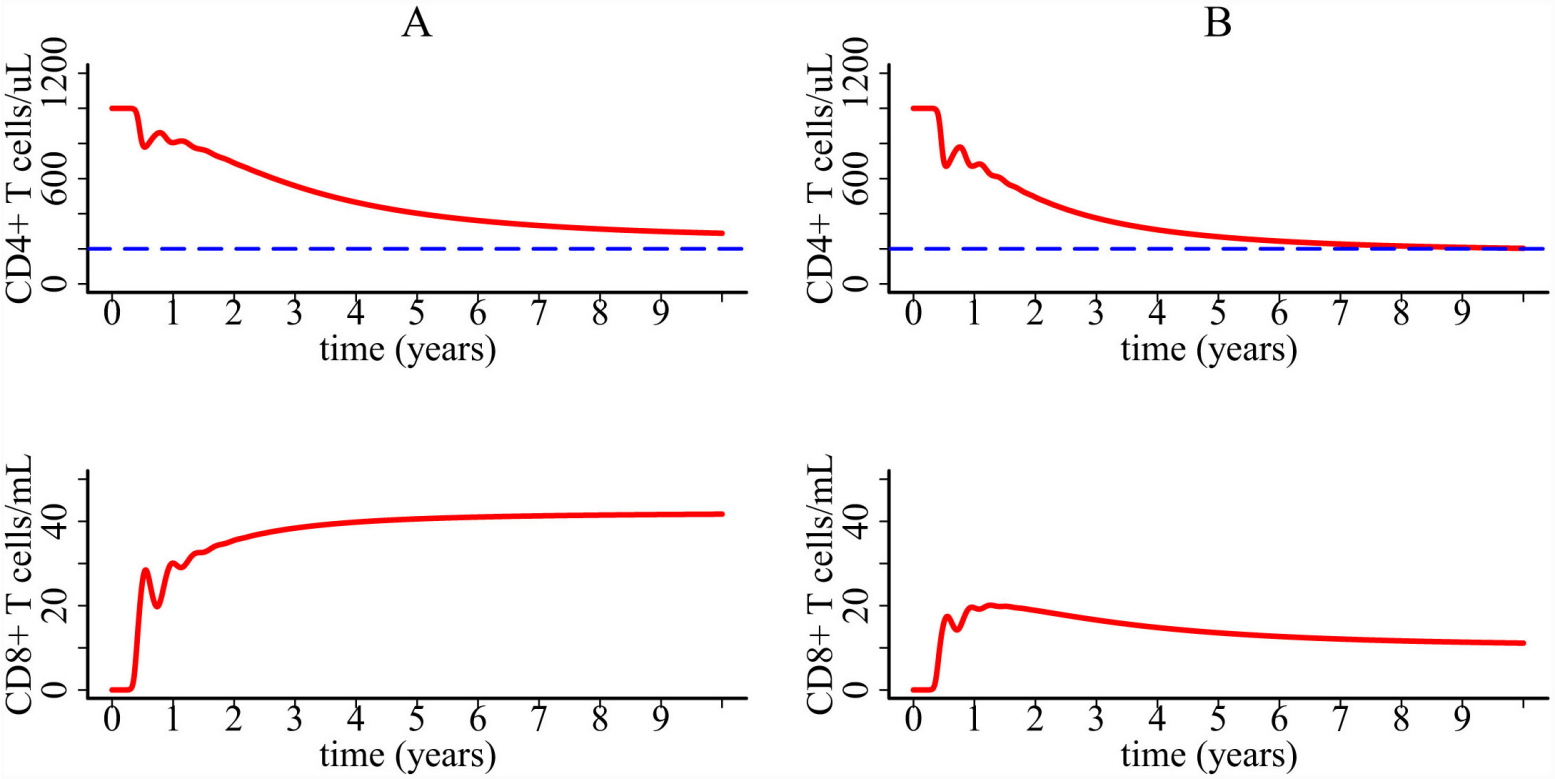
Simulation of the one-compartment model with CD8+ T cell response. Column A: predicted T cell dynamics assuming that CD8 activation is not regulated by CD4+ T cells (i.e. without the term *T*/(*T*+*η*). Column B: predicted T cell dynamics assuming that CD8 activation is regulated by CD4+ T cells. Parameter values are listed in Table 1.

The simulation of the model with CD8+ T cell response is shown in Fig 7. Parameter values are listed in Table 1. For comparison, we plotted the predicted T cell dynamics with and without the influence of CD4+ T cells. In column A of Fig 7, we performed the simulation without *T*/(*T*+*η*). CD4+ T cells decline slowly and CD8+ T cells reach a steady state level. Column B shows the simulation with the term *T*/(*T*+*η*). CD8+ T cell response becomes weaker than in column A because of the slow depletion of CD4+ T cells. CD4+ T cells decline faster because of the incapability of CD8+ T cells to control viral infection.

### Two-compartment model

**Fig 8:**
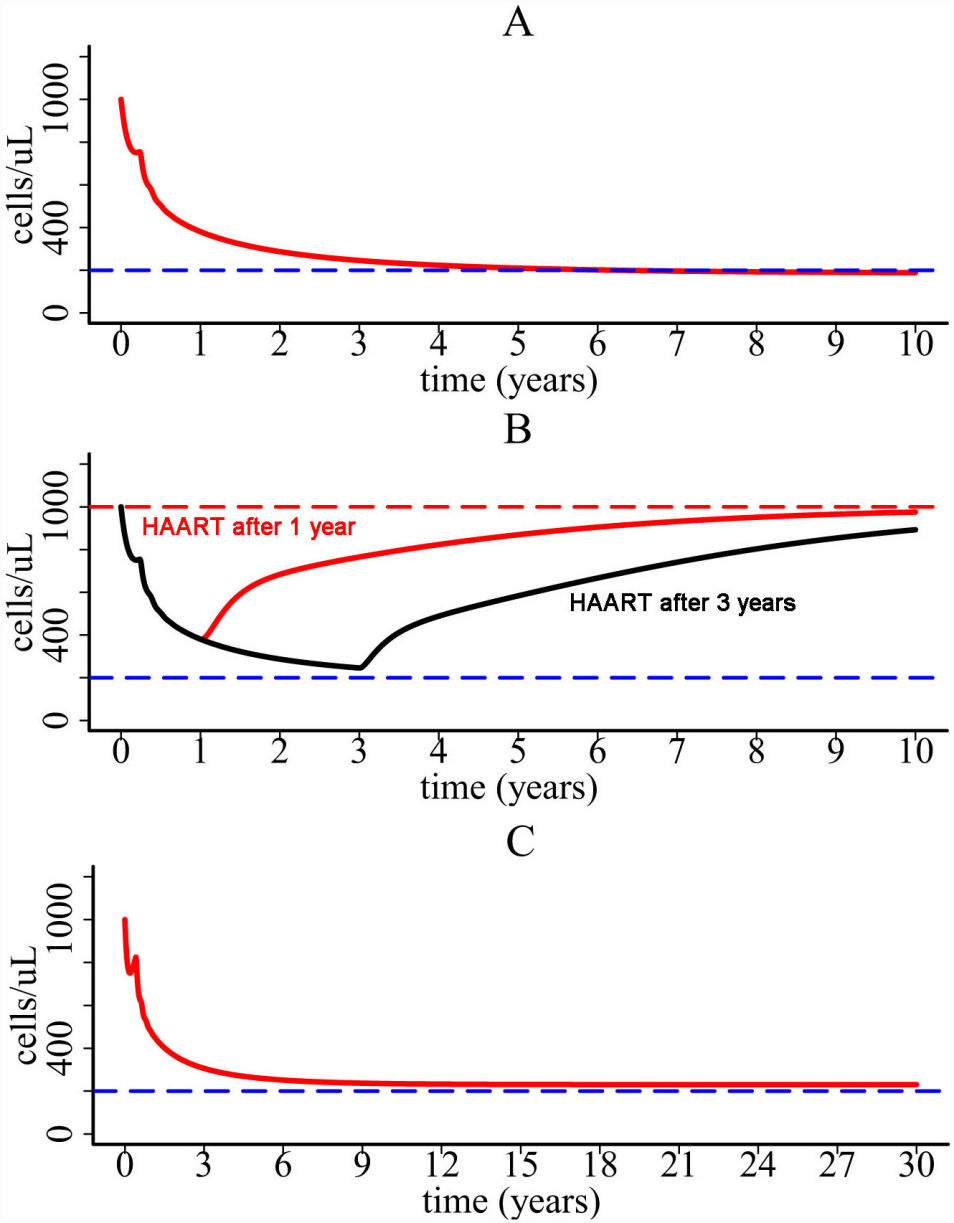
Simulation of the two-compartment model. (A) CD4+ T cell dynamics predicted by the two-compartment model. (B) Simulation of the model with *ε*_*1*_ = *ε*_*2*_ *=* 0.9. The red and black lines are the simulation when HAART is initiated 1 year and 3 years, respectively, after infection. (C) Long-term CD4+ T cell dynamics predicted with *ε*_*1*_ = 0.9 and *ε*_*2*_ = 0.4 when HAART is initiated at the beginning of infection. Because approximately 98% of viral replication takes place within lymph nodes [2,46], in the simulation we fixed *λ*_*1*_ to be 10^4^ cell ml^-1^ day^-1^ and *λ*_*2*_ to be 50 times of *λ*_*1*_. Using the equilibrium before infection, we chose the rate *σ*_*1*_ to be 50 times of *σ*_*2*_ (*σ*_*1*_ = 0.01 day^-1^ and *σ*_*2*_ = 0.0002 day^-1^). The other parameters were chosen to be *γ*_*r*_ = 5×10^−6^ ml molecule^-1^, *D*_*1*_ = 0.1 day^-1^, *D*_*2*_ = 0.2 day^-1^, *p*_*v1*_ = 1000 day^-1^, and *p*_*v2*_ = 2000 day^-1^ [32].

Inflammatory signals released during pyroptosis induce the movement of CD4+ T cells from circulation in blood to inflamed lymph nodes [43–46]. We developed a more comprehensive model by including two cell compartments (Fig 2). One is the blood compartment and the other is the compartment of lymphoid tissues where pyroptosis takes place. Simulation of the two-compartment model shows that CD4+ count in blood declines from 10^3^ cells/ul to 200 cells/ul over a long time period (Fig 8A). The viral load change in blood is also similar to that shown in Fig 3B except that T cell and viral load dynamics generated by the two-compartment model have less oscillation than by the one-compartment model.

Using the two-compartment model we also tested if HAART can rescue CD4+ T cell population. We assumed that the drug efficacies of HAART within blood and lymph node are different (i.e., the viral infection rate *k* in compartment I is reduced by 1-*ε*_*1*_ and *k* in compartment II is reduced by 1-*ε*_*2*_). We found that if the drug efficacies in both compartments are high, then CD4+ T cell depletion can be prevented (Fig 8B). The time for CD4+ restoration also depends on when HAART is initiated. However, if the drug efficacy in compartment II is relatively low (e.g. *ε*_*2*_ = 0.4) compared with the high efficacy in compartment I (e.g. *ε*_*1*_ = 0.9), then CD4+ T cells decline even when HAART is initiated at the beginning of viral infection (Fig 8C). In the simulation, CD4+ T cells stabilize at 230 cells/ul after more than 30 years (Fig 8C). This result suggests that even if some lymphoid tissues might be difficult for drug's penetration (i.e. drug sanctuary sites), CD4+ T cells can be maintained at a higher level in treated patients than in untreated patients. This may explain the increased life expectancies in HIV patients treated with combination therapy [47–51]. However, because of the CD4+ cell decline (Fig 8C), life expectancy should be lower in patients with lower baseline CD4+ cell counts than in those with higher baseline counts. This is consistent with the reported life expectancy of individuals on combination therapy in a collaborative analysis of 14 cohort studies [47].

### Comparison with long-term CD4+ T cell data

**Fig 9:**
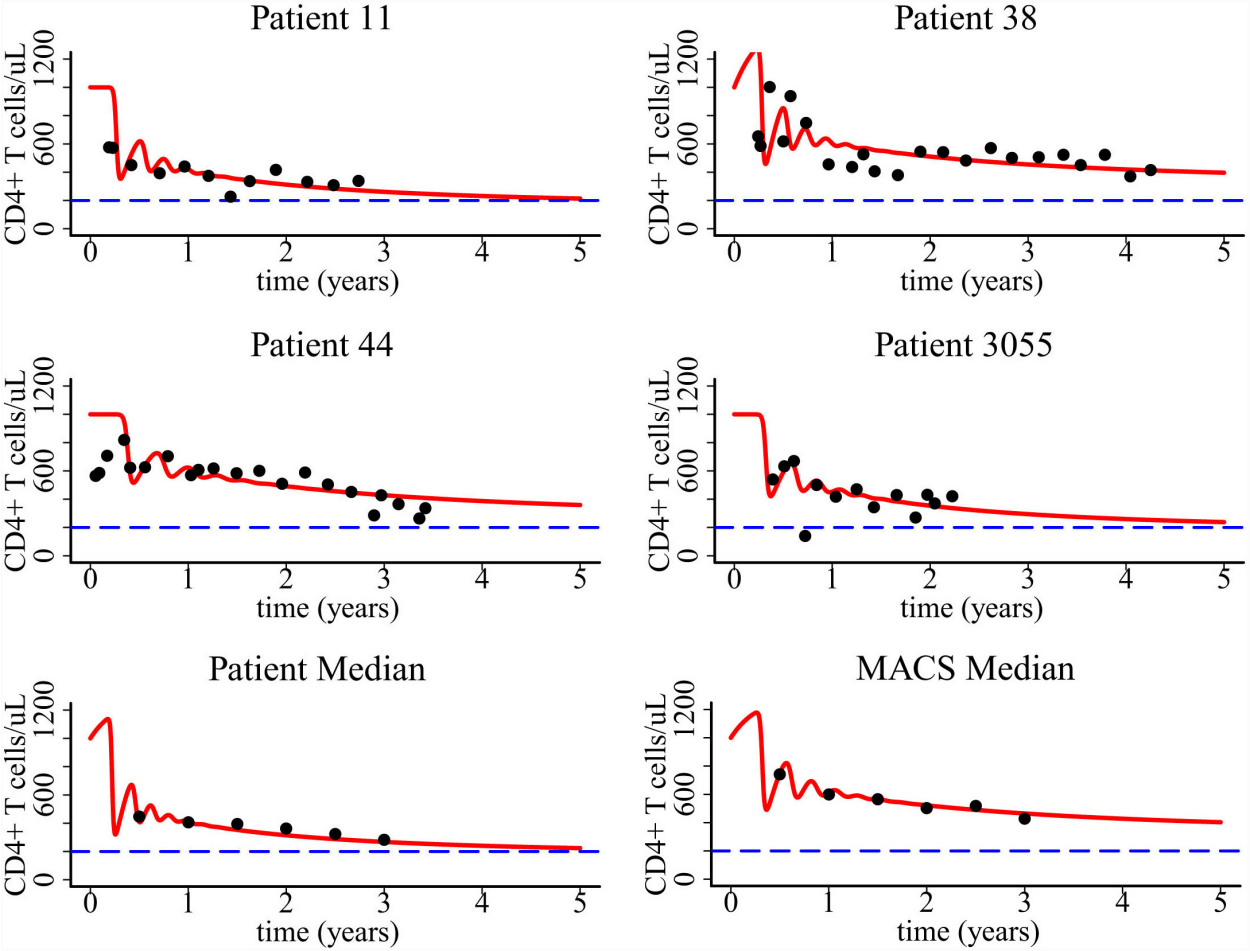
Fitting of the one-compartment model to patient data. Patient median was derived from two cohorts of studies in ref. [25] and MACS median was derived from the Multicenter AIDS Cohort Study in ref. [29]. Parameters values based on the best fits, 95% confidence intervals, and the AIC values of the fitting are listed in Table 2.

**Fig 10:**
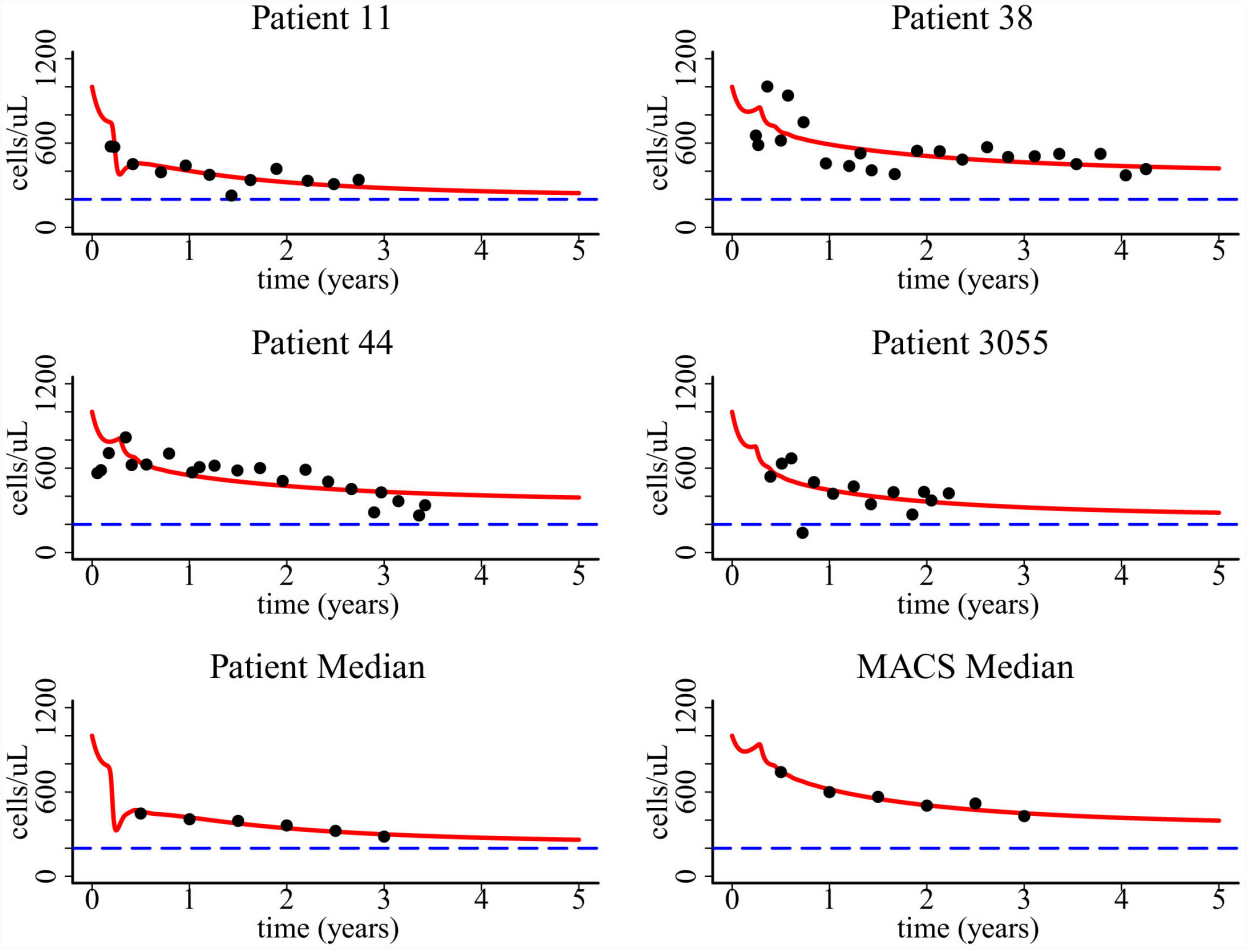
Fitting of the two-compartment model to the same patient data shown in Fig 9. Parameters values based on the best fits, 95% confidence intervals, and the AIC values of the fitting are listed in Table 3.

We compared modeling predictions with the CD4+ T cell data shown in [25–29]. Using the one-compartment model, we fit parameters *k*, *γ*_*i*_, *λ*, *p*_*v*_ and fix the other parameters for each patient. We also fit the model to the median data calculated from all the patients in the two cohort study [25] and the median data of the Current Study Multicenter AIDS Cohort Study (MACS) [29]. Using the two-compartment model, we fit parameters *k*, *γ*_*r*_, *λ*_*1*_, *p*_*v1*_ to the same patient and median data. Figs 9 and 10 show that both models provide a good fit to the long-term CD4+ T cell data in untreated HIV-1 patients. The fit to the median data is better than the fit to individual patients based on the calculated error between modeling prediction and data. These data fits suggest that pyroptosis induced CD4+ T cell movement during abortive infection can explain the progressive CD4+ T cell depletion observed in untreated HIV-1 patients.

Parameter estimates and their 95% confidence intervals based on the fits to the one-compartment and two-compartment models are listed in Tables 2 and 3, respectively. The estimate of the viral production rate *p*_*v*_ in the one-compartment model is higher than the viral production rate *p*_*v1*_ in blood of the two-compartment model (*p*_*v2*_ *=* 2000 virions per cell per day is fixed during fitting). This is because in the one-compartment model 95% of infection is assumed to be abortive and only 5% of infection produces virus. Thus, a higher value of viral production rate is needed to generate viral load with reasonable magnitude. In the second-compartment model, although only 5% of infection produces viruses in lymphoid tissues, the target cell level is much higher in lymphoid tissues than in blood (i.e. *λ*_*2*_ *>> λ*_*1*_). Thus, the viral production rates in the two compartments are on the same order of magnitude.

**Table 2:** Parameter estimates, 95% confidence intervals, and AIC values when fitting the one-compartment model to patient data.

Patient	*k* (10^−8^ *ml virion* ^*-1*^ *day* ^*-1*^)	*γ*_*i*_ (10^−4^ *ml molecule* ^*-1*^)	*λ* (10^4^ *cell ml* ^*-1*^ *day* ^*-1*^)	*p*_*v*_ (10^4^ *cell* ^*-1*^ *day* ^*-1*^)	*AIC*
11	2.4	1.5	1.0	3.0	132.7
	[1.97, 2.58]	[0.75, 1.67]	[0.81, 1.31]	[2.78, 3.12]	
38	2.3	0.5	1.5	2.6	229.7
	[2.19, 2.74]	[0.46, 0.81]	[0.55, 1.94]	[2.50, 3.20]	
44	2.1	1.0	1.0	3.0	222.2
	[2.05, 2.27]	[0.51, 1.97]	[0.81, 1.13]	[2.77, 3.09]	
3055	2.3	1.5	1.0	2.9	129.7
	[1.81, 2.67]	[1.33, 1.55]	[0.72, 1.26]	[2.42, 3.22]	
Median of patient data	2.4	1.0	1.3	3.0	47.1
	[2.33, 2.51]	[0.99, 1.17]	[1.10, 1.51]	[2.96, 3.63]	
Median of MACS data	2.0	0.6	1.3	3.0	44.3
	[1.83, 2.35]	[0.46, 0.81]	[1.11, 1.53]	[2.30, 3.10]	

**Table 3:** Parameter estimates, 95% confidence intervals, and AIC values when fitting the two-compartment model to patient data.

Patient	*k* (10^−8^ *ml virion* ^*-1*^ *day* ^*-1*^)	*γ*_*r*_ (10^−6^ *ml molecule* ^*-1*^)	*λ*_*1*_ (10^4^ *cell ml* ^*-1*^ *day* ^*-1*^)	*p*_*v1*_ (10^3^ *cell* ^*-1*^ *day* ^*-1*^)	*AIC*
11	2.3	4.0	1.0	2.0	111.1
	[1.93, 2.51]	[1.16, 4.43]	[0.66, 1.09]	[0.53, 2.67]	
38	2.0	2.0	1.2	1.0	222.4
	[1.88, 2.59]	[1.57, 8.14]	[0.66, 1.47]	[0.24, 3.75]	
44	2.0	2.0	1.1	1.0	208.9
	[1.37, 2.25]	[1.07, 4.27]	[0.88, 1.24]	[0.21, 1.28]	
3055	2.4	3.0	1.0	1.0	132.4
	[1.18, 2.66]	[1.65, 5.94]	[0.94, 1.36]	[0.42, 1.22]	
Median of patient data	2.4	4.0	1.1	2.0	41.3
	[2.33, 2.54]	[1.05, 5.35]	[0.71, 1.13]	[0.92, 2.55]	
Median of MACS data	2.0	3.0	1.4	1.0	45.3
	[1.18, 2.66]	[1.52, 3.67]	[1.20, 1.59]	[0.66, 1.09]	

The Akaike information criterion (AIC) value is calculated to compare data fitting using the two models (Tables 2 and 3). We found that for patients 11, 38, 44, and median of patient data, the AIC value of using the second model is less than that of using the first model. This suggests that the two-compartment model provides a better fit to the data for these patients from a statistical viewpoint.

### Latent reservoir persistence

IL-7 plays an important role in latently infected CD4+ T cell proliferation [52]. It has been observed to be over expressed in inflamed tissues [53,54]. Inflammatory cytokines released during cell death by pyroptosis may promote the establishment and persistence of the latent reservoir in HIV patients. Here we included the population of latently infected CD4+ T cell (*L*) into the one-compartment model. Latently infected CD4+ cells are produced with a fraction *μ* during HIV-1 infection. They can also be maintained by proliferation which is assumed to rely on the cytokine level (see the term 1+*φC* in the following equation where *φ* is fixed to 10^−2^ ml molecule^-1^). We chose the base proliferation rate *p*_*L*_ to be 0.001 day^-1^ [32], which represents a limited proliferation capacity in the absence of inflammatory cytokines. The carrying capacity of latently infected cells (*L*_*max*_) is fixed at 100 cells/ml [32]. The other parameter values are listed in Table 1. The equations of *L* and T* are given below and the other equations are the same as those in the one-compartment model.

$$\frac{dL}{dt}=\mu \left(1-f\right)k\left[1+\gamma_{i}C\right]VT+p_{L}\left(1+\varphi C\right)L\left(1-\frac{L}{L_{max}}\right)-d_{L}L$$

$$\frac{dT^{*}}{dt}=\left(1-f\right)\left(1-\mu \right)k\left[1+\gamma_{i}C\right]VT-d_{2}T^{*}$$

**Fig 11:**
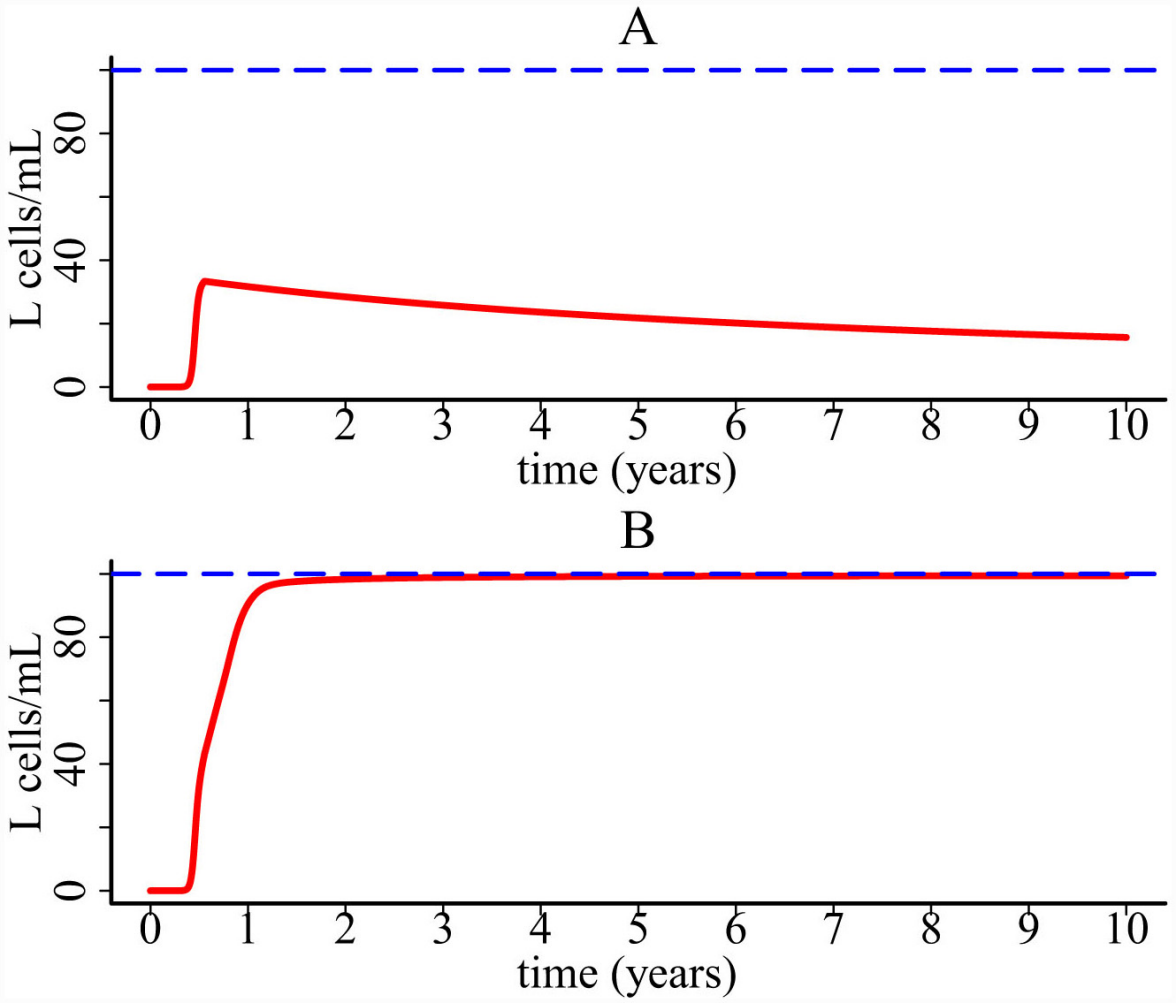
Simulation of the one-compartment model with latently infected cells. (A) Population of latently infected T cells without cytokine enhanced homeostatic proliferation. (B) Population of latently infected T cells with cytokine enhanced homeostatic proliferation.

If there is no chronic inflammation (i.e. *φ =* 0 in the *L* equation), then latently infected cells undergo a slow decline (Fig 11A). However, if the proliferation is enhanced by cytokines released during cell death by pyroptosis, then the latent reservoir can be maintained at a higher level (Fig 11B). This result suggests that inflammatory cytokines generated during abortive infection might contribute to the establishment of the latent reservoir and the maintenance of its size. We also performed sensitivity test of latently infected cells on the parameter *φ*, the effectiveness of cytokines promoting latently infected cell proliferation. The modeling prediction is robust to this parameter (S6 Fig).

Latently infected cells can be activated by relevant antigens and become productively infected cells. In S1 Text, we included the activation of latently infected cells in the one-compartment model. Simulation with different values of the activation rate is shown in S8 Fig. As the activation rate increases, the size of the latent reservoir decreases.

## Discussion

The mechanisms underlying the slow time scale of CD4+ T cell decline in untreated HIV-1 patients remain unclear. HIV-mediated cell death can contribute to the loss of CD4+ T cells, but quantitative image analysis suggested that infection-induced cell death could be compensated by upregulated T cell division [55,56]. Some studies suggested that the destruction of bystander non-infected cells may account for the CD4+ T cell decline during disease progression [57–60]. Immune activation might be the reason of bystander cellular demise [60]. It drives uninfected CD4+ T cells into several rounds of division and cells are susceptible to activation-induced death [61,62]. However, a mathematical model showed that the decline of CD4+ T cells would be very rapid if immune activation drives T cell depletion [20]. Another possible reason of T cell decline might be the regeneration failure of CD4+ T cells during disease progression [3,11–14]. A recent study found that about 95% of CD4+ T cells within lymph nodes die from pyroptosis and release inflammatory signals that attract more CD4+ T cells from elsewhere to be infected [23]. HIV-1 may use this vicious infection cycle to promote disease progression and chronic T cell depletion.

In this paper, we developed mathematical models to explore whether cell death induced by pyroptosis can explain the slow time scale of CD4+ T cell decline in untreated HIV patients. In the first model, we assumed that increased availability of target cells due to attraction by inflammatory cytokines facilitates viral infection, which drains the CD4+ T cell population slowly during chronic infection. In the second model, we explicitly included the movement of CD4+ T cells from blood to lymphoid tissues where pyroptosis occurs. Both models generate a very slow decline of CD4+ T cells in plasma (Figs 3 and 8), and agree with the long-term CD4+ T cell data from untreated HIV patients in several cohorts in Brazil (Figs 9 and 10).

We found that the entire CD4+ T cell decline consists of two major phases (Fig 3). The first-phase decline is very rapid. This decline is due to the enormous virus infection and virus-induced cell death during primary infection. Following the first phase, CD4+ T cells partially recover because of cell regeneration and viral control by immune responses. However, a balance cannot be established between cell generation and viral infection. Chronic inflammatory cytokines released during pyroptosis can attract CD4+ T cells from other places to inflamed lymphoid tissues. These cells are infected and die, resulting in a slow decline of CD4+ T cells in plasma. These results suggest that HIV-mediated cell death causes the dramatic decline of CD4+ T cells during primary infection and that persistent chronic inflammation acts like an erosive force which gradually drains the CD4+ T cell population in plasma during chronic infection. HAART was shown to have the potential to restore the CD4+ T cell population (Figs 6 and 8), which agrees with the robust and sustained CD4 recovery among patients remaining on therapy [63] and a normal life expectancy in patients with a good CD4 response and undetectable viral load [50]. However, CD4 response depends on the effectiveness of the therapy, when the therapy is initiated, and whether there exist drug sanctuary sites (Figs 6 and 8). This may explain the considerable variability in the increase of life expectancy in patients treated with combination therapy between 1996 and 2005 [47].

Our model has limitations. First, it does not account for the spatial effect of CD4+ T cells. Although we used a two-compartment model to describe the transportation of cells and virus between blood and lymphoid tissues, release of cytokines during cell death by pyroptosis and attraction of CD4+ T cells are mainly constrained to occur locally. Ordinary differential equation models could not capture these features. It would be valuable to develop spatial models that can describe the vicious cycle within lymphoid tissues. Spatial models require precise description and parameterization of diffusion of cytokines and attraction of CD4+ T cells, and are also computationally demanding in studying T cell dynamics within blood and different lymphoid tissues. The second limitation of our model is that we did not consider a detailed inflammatory signal transduction cascade between T cells and relevant tissues. Recruitment of T cells to the inflamed tissue goes through several steps of immunological reaction. Upon secretion of IL-1β, expression of adhesion molecules such as E/P-selectin and ICAM-1 on the vascular endothelium is upregulated [64]. Binding to these molecules facilitates T cell's attachment to vascular endothelium. After attachment T cells undergo conformational changes and penetrate into the inflamed tissue [65,66]. In our models, we used a very simple factor multiplied by the concentration of cytokines to describe the effect of inflammatory cytokines. A more comprehensive model requires a detailed description of intracellular processes underlying the inflammatory signal cascade and related data for model verification. The third limitation is that our model cannot generate viral load explosion in the later stages of HIV infection. Assuming that all parameters are constant and that only one cell population produces virus, our model cannot describe viral explosion. However, as CD4+ T cells drop to very low levels, the immune system cannot kill infected cells or neutralize virus effectively. This leads to a reduction in the death rate of infected cells or viral clearance rate, and may explain the viral explosion. Infection of other cell populations such as macrophages (as suggested by Hernandez-Vargas and Middleton in ref. [21]) or other viral reservoirs may also explain the dramatic viral load increase during the AIDS stage.

Our simulation shows that the latent reservoir may be maintained by chronic inflammation. How inflammation promotes the latent reservoir persistence is not fully understood. Some results suggested that caspase-1 can promote cellular survival. For example, epithelial cells activate caspase-1 to enhance membrane repair in response to the pore-forming toxins to prevent proteolysis [67]. Whether latently infected T cells can use this caspase-1 pathway to promote their survival remains unknown. Another possibility is through the dysregulated action of IL-7 or IL-15 that can stimulate homeostatic proliferation of latently infected cells. Stromal cells are located in secondary lymph organs such as lymph node trabeculae, lymph vessels, and conduits [68]. IL-7 is observed to be significantly expressed by stromal cells within inflamed lymph nodes [69]. It would be valuable to explore whether HIV-1 can use the caspase-1 pathway to persist in latent cells and whether IL-7 production can be inhibited in the inflamed microenvironment.

The results suggest that cell death by pyroptosis plays an important role in driving slow CD4+ T cell depletion. If pyroptosis can be inhibited, then CD4+ T cells might be maintained. VX-765 is a caspase-1 inhibitor [70–73] used to treat chronic epilepsy and psoriasis. It was found to be safe and well tolerated in humans in a phase IIa trial of epilepsy [74]. Doitsh et al. showed that VX-765 can inhibit secretion of IL-1β and also block cleavage of caspase-1 in HIV-infected tonsillar and splenic lymphoid tissues [23]. However, the active form of VX-765 cannot effectively inhibit cell death, which may be due to reduced cellular permeability [70]. It remains unclear whether the pro-drug VX-765 can efficiently block cell death in vivo. We showed that if antiretroviral drugs cannot effectively block viral replication in lymphoid tissues, then HIV-1 can still establish chronic inflammation in these sites. This is consistent with the observation of persistent inflammation in patients under long-term antiretroviral treatment [75,76]. When drug sanctuary sites exist, CD4+ T cells undergo a very slow depletion or stabilize at a low level (Fig 8). In this case, the immune system would be vulnerable to various opportunistic infections and neoplasms. This may explain the shorter extension of life expectancy in treated patients who had a low CD4+ cell nadir [47–49,51]. If antiretroviral drugs and caspase-1 inhibitors can be effectively delivered to human lymphoid tissues via some transporters [77,78], then CD4+ T cell depletion might be prevented and life expectancy of treated patients might be further extended.

## Supporting Information

S1 Text Additional models and sensitivity test. (DOCX)

S1 Fig CD4+ T cells predicted by the one-compartment model with target cell proliferation. The proliferation rate *p* is chosen to be 0.001 day^-1^ (red), 0.01 day^-1^ (blue), 0.1 day^-1^ (black), and 1 day^-1^ (brown) in the simulation.
(EPS)

S2 Fig Sensitivity test of CD4+ T cells on the cytokine burst size *N*_*c*_. The value of *N*_*c*_ increases from 9 molecules (orange line) to 21 molecules (black line) with four equal increments.
(EPS)

S3 Fig Sensitivity test of CD4+ T cells on the death rate (*d*_*3*_) of abortively infected cells. The value of *d*_*3*_ increases from 0.0006 day^-1^ (orange line) to 0.0014 day^-1^ (black line) with four equal increments.
(EPS)

S4 Fig Sensitivity test of CD4+ T cells on the cytokine decay rate *d*_*5*_. The value of *d*_*5*_ increases from 3.96 day^-1^ (orange line) to 9.24 day^-1^ (black line) with four equal increments.
(EPS)

S5 Fig Sensitivity test of CD4+ T cells on the fraction (*f*) of abortive infection. The value of *f* increases from 93.1% (orange line) to 96.9% (black line) with four equal increments.
(EPS)

S6 Fig Sensitivity test of latently infected cells on the effect (*φ*) of cytokines promoting latently infected cell proliferation. The value of *φ* increases from 0.006 ml molecule^-1^ (orange line) to 0.014 ml molecule^-1^ (black line) with four equal increments.
(EPS)

S7 Fig CD4+ T cells predicted by the two-compartment model with transportation of productively infected cells between compartments. In the simulation, the value of *D*_*1*_ * is fixed to 0.2 day^-1^ and *D*_*2*_ * is fixed to 0.1 day^-1^.
(EPS)

S8 Fig Simulation of latently infected cells with different rates of activation *a*_*L*_. The value of *a*_*L*_ increases from 0.01 day^-1^ (red line) to 0.05 day^-1^ (orange line) with four equal increments.
(EPS)
