# Supplementary material for: Modeling the Slow CD4+ T Cell Decline in HIV-Infected Individuals
Source: PLoS Comput Biol. 2015 Dec 28;11(12):e1004665. doi: 10.1371/journal.pcbi.1004665 (PMC4692447; doi:10.1371/journal.pcbi.1004665)
Supplement: S1 Text — (DOCX) [file pcbi.1004665.s001.docx]

**S1 Text. Additional models and sensitivity test**

**1. Model with target cell proliferation**

We simulated the case in which target cells are assumed to proliferate. The proliferation is modeled by a logistic term $pT(1-\frac{T}{T_{max}})$ where *p* is the maximum proliferation rate and *T_max_* is the carrying capacity of CD4+ T cells. The *T* equation in the one-compartment model becomes

$$\frac{dT}{dt}=\lambda+pT(1-\frac{T}{T_{max}})-k(1+\gamma_{i}C)VT-d_{1}T$$

Simulation with different proliferation rates is shown in S1 Fig. As the proliferation rate increases, the decline of CD4+ T cells becomes faster. This is because more target cells lead to more abortive infection, which releases more cytokines attracting more CD4+ T cells to be infected and die.

**2. Sensitivity test**

In addition to sensitivity tests shown in Fig 4, we performed more analysis of the sensitivity of our modeling prediction on parameters *N*_c_, *d_3_*, *d_5_*, *f* and *φ*, which is shown in S2 Fig - S6 Fig, respectively.

**3. Model with transportation of infected cells between compartments**

We studied the impact of including transportation of productively infected T cells between blood and lymph node on T cell dynamics. Similar to viral transfer between compartments, we assumed that infected cells (*T_1_^*^* and *T_2_^*^*) can transport between two compartments at rate *D_1_^*^*(*T_2_^*^*-*T_1_^*^*) and *D_2_^*^*(*T_1_^*^*-*T_2_^*^*). The productively infected cell equations become

$$\frac{dT_{1}^{*}}{dt}=kV_{1}T_{1}-d_{2}T_{1}^{*}+D_{1}^{*}(T_{2}^{*}-T_{1}^{*})$$

$$\frac{dT_{2}^{*}}{dt}=\left( 1-f \right)kV_{2}T_{2}-d_{2}T_{2}^{*}+D_{2}^{*}(T_{1}^{*}-T_{2}^{*})$$

In the simulation (S7 Fig), the value of *D_1_^*^* is fixed to 0.2 day^-1^ and *D_2_^*^* is fixed to 0.1 day^-1^. We found that including the transportation of productively infected T cells between compartments has a minor effect on T cell dynamics (S7 Fig). Because the dynamics of virus are much faster than those of cells, it is reasonable to assume that they are proportional to each other. Thus, in the main text we only included the transportation of virus between compartments.

**4. Model with latently infected cell activation**

We assumed that latently infected cells (*L*) can be activated by antigens and become productively infected cells ($T^{*}$) at rate *a_L_*. Thus, the *L* and $T^{*}$ equations become the following. The other equations remain unchanged.

$$\frac{dL}{dt}=\mu\left( 1-f \right)k\left[ 1+\gamma_{i}C \right]VT+p_{L}\left( 1+\varphi C \right)L\left( 1-\frac{L}{L_{max}} \right)-d_{L}L-a_{L}L$$

$$\frac{dT^{*}}{dt}=\left( 1-f \right)\left( 1-\mu\right)k\left[ 1+\gamma_{i}C \right]VT-d_{2}T^{*}+a_{L}L$$

Simulation with different values of the activation rate *a_L_* is shown in S8 Fig. As the activation rate increases, the size of the latent reservoir decreases.
